# Supplementary material for: Proteomic Analysis Comparison on the Ecological Adaptability of Quinclorac-Resistant Echinochloa crus-galli
Source: Plants (Basel). 2023 Feb 4;12(4):696. doi: 10.3390/plants12040696 (PMC9968053; doi:10.3390/plants12040696)
Supplement: Supplementary file 1 [file plants-12-00696-s001.zip › Supplementary Materialsú║the method of protein extraction, digestion.pdf]

1.1 Protein extraction

1. Frozen samples were ground to powders;
2. The sample powders were transferred into low protein binding tubes (1.5ml Eppendorf);
3. The extraction buffer were added to the samples for 1ml and mixed.
4. The mixtures were added with Tris-phenol buffer and mixed for 30min at 4°C.
5. The mixtures were centrifuged at 7100g for 10min at 4°C to collect phenol supernatants.
6. The supernatants were added for 5 volumes of 0.1M cold ammonium acetate - methanol buffer and precipitated at -20°C overnight.
7. The samples were centrifuged at 12000g for 10min to collect precipitations.
8. The precipitations were washed by 5 volumes of cold methanol and gently mixed.
9. The samples were centrifuged at 12000g for 10min at 4°C to collect precipitations. Repeat once.
10. The methanol was replaced by acetone and repeat Step 8 and 9 twice to remove methanol contamination.
11. Then the samples were centrifuged at 12000 g for 10 min at 4°C to collect precipitations.
12. The precipitations were dried at room temperature for about 3min and dissolved in lysis buffer for 2hrs.
13. The samples were centrifuged at 12000g for 10min to collect supernatants. The supernatants were centrifuged again to remove precipitations completely.
14. Protein concentration was determined by Bradford assay and aliquoted to store at -80° C.

1.2 Measurement of protein

Measurement of protein were performed by BCA method.

1.3 SDS-PAGE electrophoresis

1. The 15ug proteins of each sample were acquired and separated by 12% SDS-PAGE gel.
2. The separation gel was stained by CBB according to Candiano's protocol:

A. The gel was fixed for 2 h.

B. Then the gel was stained for 12 h.

C. After staining, the gel was washed with water until the bands were visualized.

3. The stained gel was scanned by ImageScanner (GE Healthcare, USA) at the resolution of 300dpi.

1.4 Protein digestion and iTRAQ labeling

1. 100 μg protein extraction was subjected with 120 μL reducing buffer (10 mM DTT, 8 M Urea, 100 mM TEAB, pH 8.0) on 10K ultrafiltration tube. And the solution was incubated at 60°C for 1 h.
2. IAA was added to the solution with the final concentration of 50 mM in the dark at room temperature for 40 min.
3. The solutions were centrifuged on the filters at 12000 rpm for 20 min at 4°C. The flow-through were discarded from the collection tube.
4. 100 μL 100 mM TEAB was added to the solutions and centrifuged at 12000 rpm for 20 min. Repeat twice.
5. The filter units were transferred into new collection tubes. 100 μL 100 mM TEAB was added and followed with 2 μl sequencing-grade trypsin (1 μg/μL) in each tube, then the solutions were incubated for digestion at 37°C for 12 h.
6. The collections of digested peptides were centrifuge at 12000 rpm for 20 min. 50 μL 100 mM TEAB were added and centrifuged again. Mix up the collected solution. The solutions were collected and lyophilized.
7. The lyophilized samples were resuspended in 100 μL 50 mM TEAB and 40 μL of each sample were transferred into new tubes for labeling.

8. 200 μL isopropanol was added to iTRAQ reagent vial at room temperature. The centrifuged reagents were dissolved for 5 min and mixed for centrifugation. Repeat once.
9. 100 μL of the iTRAQ label reagent was added to each sample for mixing. The tubes were incubated at room temperature for 2 h.
10. 200 μL of HPLC water was added to each sample and incubated for 30 min to terminate reaction. The labeling peptides solutions were lyophilized and stored at -80°C.
